# PRODUCTIVE ACTIVITIES AND LONELINESS AMONG JAPANESE MIDLIFE AND OLDER ADULTS

**DOI:** 10.1093/geroni/igz038.3049

**Published:** 2019-11-08

**Authors:** Yang Wang, Yihan Wang, Florian Kohlbacher, Ernest Gonzales

**Affiliations:** 1 University of Illinois at Urbana-Champaign, Urbana, Illinois, United States; 2 Boston College, Boston, Massachusetts, United States; 3 The Economist Intelligence Unit, Economist Corporate Network, North Asia, The Economist Group, Tokyo, Japan; 4 New York University, Silver School of Social Work, New York, New York, United States

## Abstract

Background: Loneliness among older population is a public health concern shared worldwide. Using the motivational theory for life-span development, this study examined the associations between loneliness (social and emotional) and productive activities among midlife and older adults in Japan. Methods: The Japanese National Data on Lifestyle and Mental Health, a nationally representative sample of midlife and older adults (2011, N=1,575), were used to examine how employment, volunteering, helping family and friends, and informal caregiving was associated with social and emotional loneliness, controlling for multiple risk and protective factors. Results: Family caregiving was related to more social loneliness. Working, helping family, and volunteering were related to less emotional loneliness, while family caregiving was related to more emotional loneliness. Japanese male caregivers reported more social isolation compared to female caregivers. Face-to-face interactions reduced emotional loneliness among caregivers. Discussion: Findings underscored the nuanced difference of social loneliness and emotional loneliness. Social policies that advance productive aging should recognize “unintended consequences” and aim to protect older adults from social and emotional loneliness. Counselling services and social support programs specifically for Japanese male caregivers are warranted.

